# Preliminary Incidence and Trends of Infections Caused by Pathogens Transmitted Commonly Through Food — Foodborne Diseases Active Surveillance Network, 10 U.S. Sites, 2022

**DOI:** 10.15585/mmwr.mm7226a1

**Published:** 2023-06-30

**Authors:** Miranda J. Delahoy, Hazel J. Shah, Daniel Lowell Weller, Logan C. Ray, Kirk Smith, Suzanne McGuire, Rosalie T. Trevejo, Elaine Scallan Walter, Katie Wymore, Tamara Rissman, Marcy McMillian, Sarah Lathrop, Bethany LaClair, Michelle M. Boyle, Stic Harris, Joanna Zablotsky-Kufel, Kennedy Houck, Carey J. Devine, Carey E. Lau, Robert V. Tauxe, Beau B. Bruce, Patricia M. Griffin, Daniel C. Payne

**Affiliations:** ^1^Division of Foodborne, Waterborne, and Environmental Diseases, National Center for Emerging and Zoonotic Infectious Diseases, CDC; ^2^Minnesota Department of Health; ^3^New York State Department of Health; ^4^Oregon Health Authority; ^5^Colorado Department of Public Health and Environment; ^6^California Emerging Infections Program, Oakland, California; ^7^Connecticut Emerging Infections Program, New Haven, Connecticut; ^8^Tennessee Department of Health; ^9^University of New Mexico, Albuquerque, New Mexico; ^10^Georgia Department of Public Health; ^11^Maryland Department of Health; ^12^Center for Food Safety and Applied Nutrition, Food and Drug Administration, College Park, Maryland; ^13^Food Safety and Inspection Service, U.S. Department of Agriculture, Washington, DC.

Each year, infections from major foodborne pathogens are responsible for an estimated 9.4 million illnesses, 56,000 hospitalizations, and 1,350 deaths in the United States (*1*). To evaluate progress toward prevention of enteric infections in the United States, the Foodborne Diseases Active Surveillance Network (FoodNet) conducts surveillance for laboratory-diagnosed infections caused by eight pathogens transmitted commonly through food at 10 U.S. sites. During 2020–2021, FoodNet detected decreases in many infections that were due to behavioral modifications, public health interventions, and changes in health care–seeking and testing practices during the COVID-19 pandemic. This report presents preliminary estimates of pathogen-specific annual incidences during 2022, compared with average annual incidences during 2016–2018, the reference period for the U.S. Department of Health and Human Services’ Healthy People 2030 targets (*2*). Many pandemic interventions ended by 2022, resulting in a resumption of outbreaks, international travel, and other factors leading to enteric infections. During 2022, annual incidences of illnesses caused by the pathogens *Campylobacter*, *Salmonella*, *Shigella*, and *Listeria* were similar to average annual incidences during 2016–2018; however, incidences of Shiga toxin-producing *Escherichia coli* (STEC), *Yersinia*, *Vibrio*, and *Cyclospora* illnesses were higher. Increasing culture-independent diagnostic test (CIDT) usage likely contributed to increased detection by identifying infections that would have remained undetected before widespread CIDT usage. Reducing pathogen contamination during poultry slaughter and processing of leafy greens requires collaboration among food growers and processors, retail stores, restaurants, and regulators.

CDC, 10 state health departments, the U.S. Department of Agriculture’s Food Safety and Inspection Service (FSIS), and the Food and Drug Administration (FDA) collaborate to conduct active population-based surveillance of the FoodNet catchment area,* which included an estimated 51 million persons in 2022 (approximately 15% of the U.S. population). Laboratories diagnose bacterial infections by culture or CIDT and *Cyclospora* infections by microscopy or polymerase chain reaction.^†^ Infection incidence was calculated by dividing the number of infections during 2022 by 2021 U.S. Census Bureau population estimates for the surveillance area and is reported as infections per 100,000 persons. A Bayesian, negative binomial model with penalized thin plate splines adjusting for state-specific trends and population changes^§^ was used to estimate incidence changes during 2022 compared with the average annual incidence during 2016–2018 using the brms package (version 2.14.0) in R software (version 3.6.2, R Foundation).^¶^ Incidence was described as increased or decreased relative to the reference period if the 95% credible interval (CrI) for the incidence rate ratio (IRR) did not cross the null value of 1. Incidence changes were also estimated using this method for the subset of infections that were domestically acquired.** Frequencies of hospitalizations, deaths, outbreak-associated infections, and international travel-associated infections were calculated overall and by pathogen.^††^ The proportion of infections that were diagnosed by CIDT^§§^ and diagnosed only by CIDT (meaning the specimen had a negative culture result or was not cultured), the proportion of infections diagnosed by CIDT for which a culture was performed, and the proportion of those cultures yielding an isolate were calculated by pathogen for bacterial infections.

A network of nephrologists and infection preventionists conducts surveillance for diagnosed pediatric post-diarrheal hemolytic uremic syndrome (HUS), a complication of STEC infection that most commonly occurs among young children; additional HUS data are collected by hospital discharge review.^¶¶^ This report includes HUS cases and incidence per 100,000 children and adolescents aged <18 years detected during 2021, the most recent year with available data. This activity was reviewed by CDC and conducted consistent with applicable federal law and CDC policy.***

During 2022, FoodNet identified 25,479 cases of infection, 5,981 hospitalizations, and 170 deaths (Table 1). Infection incidence was highest for *Campylobacter* (19.2 cases per 100,000 population), followed by *Salmonella* (16.3). Compared with pathogen-specific average annual incidences during 2016–2018, STEC, *Yersinia*, *Vibrio*, and *Cyclospora* infection incidences were higher during 2022. Overall infection incidence was stable for *Campylobacter*, *Salmonella*, *Shigella*, and *Listeria.* However, when limited to domestically acquired infections, *Campylobacter* incidence was higher during 2022 (IRR = 1.07, 95% CrI = 1.01–1.14), as were incidences for *Yersinia, Vibrio,* and *Cyclospora*. Compared with 2016–2018, similar percentages of infections during 2022 resulted in hospitalization (23.5% in 2022 versus 23.8%) and death (0.7% versus 0.5%) or were associated with outbreaks (4.3% versus 3.9%) or international travel (12.4% versus 12.8%). However, 62 *Salmonella* infections (0.7%) resulted in death during 2022, compared with an annual average of 37 (0.4%) during 2016–2018. Serotypes and characteristics of *Salmonella* infections resulting in death were similar to those during 2016–2018 (FoodNet, unpublished data, 2023).^†††^

**TABLE 1:** Number of laboratory-diagnosed bacterial and parasitic infections, hospitalizations, deaths, outbreak-associated infections, crude incidence, and incidence rate ratios compared with 2016–2018 average annual incidence, domestic incidence, and Healthy People 2030 incidence targets,* by pathogen — Foodborne Diseases Active Surveillance Network, 10 U.S. sites,^†^ 2022^§^

Pathogen	Infections,^¶^ no.	No. (%)			Crude average incidence 2016–2018	Crude incidence 2022^¶¶^	IRR (95% CrI)***	Domestic incidence^†††^	Healthy People 2030 (domestic) incidence target
		Hospitalizations**	Deaths^††^	Outbreak-associated infections^§§^					
**Bacteria**									
*Campylobacter*	9,751	1,938 (19.9)	42 (0.4)	59 (0.6)	18.8	19.2	1.02 (0.96–1.08)	17.4	10.9
*Salmonella*	8,285	2,228 (26.9)	62 (0.7)	756 (9.1)	17.0	16.3	0.95 (0.89–1.02)	14.5	11.5
STEC^§§§^	2,882	582 (20.2)	11 (0.4)	78 (2.7)	5.3	5.7	1.18 (1.02–1.36)	4.6	3.7
STEC O157^¶¶¶^	301	—****	—****	—****	0.9	0.6	0.76 (0.65–0.86)	—****	NA^††††^
STEC non- O157^¶¶¶^	992	—****	—****	—****	2.1	2.0	0.92 (0.77–1.13)	—****	NA^††††^
*Shigella*	2,478	758 (30.6)	6 (0.2)	136 (5.5)	5.1	4.9	0.95 (0.75–1.18)	3.9	NA^††††^
*Yersinia*	1,003	200 (19.9)	5 (0.5)	6 (0.6)	0.9	2.0	2.41 (2.03–2.88)	1.9	NA^††††^
*Vibrio*	504	117 (23.2)	13 (2.6)	0 (—)	0.8	1.0	1.57 (1.37–1.81)	0.9	NA^††††^
*Listeria* ^§§§§^	136	128 (94.1)	30 (22.1)	7 (5.1)	0.3	0.3	1.06 (0.93–1.22)	0.26	0.22
**Parasite**									
*Cyclospora*	440	30 (6.8)	1 (0.2)	54 (12.3)	0.4	0.9	4.77 (2.60–10.7)	0.6	NA^††††^
**Total**	**25,479**	**5,981 (23.5)**	**170 (0.7)**	**1,096 (4.3)**	—****	—****	—****	—****	—****

**Abbreviations**: CIDT = culture-independent diagnostic test; CrI = credible interval; HHS = U.S. Department of Health and Human Services; IRR = incidence rate ratio; NA = not applicable; STEC = Shiga toxin-producing *Escherichia coli*.

* Healthy People 2030 is a 10-year plan for addressing critical public health priorities and challenges. HHS releases priority objectives as part of this plan, including incidence targets for select causes of foodborne illness (resulting from *Campylobacter*, *Salmonella*, STEC, and *Listeria*), to be met by 2030. https://health.gov/healthypeople/objectives-and-data/browse-objectives/foodborne-illness

^†^ Data were obtained from laboratories in Connecticut, Georgia, Maryland, Minnesota, New Mexico, Oregon, Tennessee, and selected counties in California, Colorado, and New York.

^§^ 2022 data are preliminary.

^¶^ Bacterial infections were diagnosed using culture or CIDT. *Cyclospora* infections were diagnosed using microscopy or polymerase chain reaction.

** Admission to an inpatient unit or an observation stay of >24 hours within 7 days before or after specimen collection or determined to be related to the infection if beyond this time frame. Average percentage of infections resulting in hospitalizations during 2016–2018 by pathogen: *Campylobacter* (20%), *Salmonella* (27%), STEC (22%), *Shigella* (24%), *Yersinia* (26%), *Vibrio* (30%), *Listeria* (96%), *Cyclospora* (6%), and overall (24%). Infections with unknown hospitalization status (8% of infections during 2022 and 4% during 2016–2018) were included in the denominator only (i.e., classified as not hospitalized).

**^††^** Attributed to infection when death occurred during hospitalization or within 7 days after specimen collection from nonhospitalized patients. Average percentage of infections resulting in death during 2016–2018 by pathogen: *Campylobacter* (0.4%), *Salmonella* (0.4%), STEC (0.4%), *Shigella* (0.1%), *Yersinia* (1.2%), *Vibrio* (2.1%), *Listeria* (18.6%), *Cyclospora* (0.2%), and overall (0.5%). Infections with unknown death status (9% of infections during 2022 and 3% during 2016–2018) were included in the denominator. *Salmonella* deaths occurred in nine of 10 surveillance sites. Among the 32 *Salmonella* deaths with information on travel, two (6%) were associated with international travel. Six *Salmonella* deaths were associated with outbreaks.

^§§^ Generally defined as ≥2 cases of similar illness associated with a common exposure; some sites also stipulate illnesses be from more than one household. Average percentage of outbreak-associated infections during 2016–2018 by pathogen: *Campylobacter* (<1%), *Salmonella* (7%), STEC (4%), *Shigella* (5%), *Yersinia* (<1%), *Vibrio* (4%), *Listeria* (5%), *Cyclospora* (24%), and overall (4%).

^¶¶^ Cases of infection per 100,000 population. Crude incidence is unadjusted and includes both infections among those who reported international travel before illness began (30 days for *Listeria* and *Salmonella* serotypes Typhi and Paratyphi, 14 days for *Cyclospora*, and 7 days for other pathogens) and domestically acquired infections (those for which the patient had no history of international travel or unknown travel history).

*** A Bayesian, negative binomial model with penalized thin plate splines adjusting for state-specific trends and population changes was used to estimate the percentage change in incidence during 2022 compared with the average annual incidence during 2016–2018. Incidence is described as increased or decreased relative to the reference period if the 95% CrI for the IRR did not cross the null value of 1. This model is based on crude incidence (i.e., includes both domestically acquired infections and those infections associated with international travel).

**^†††^** Domestic incidence refers to the incidence of domestically acquired infections. Healthy People 2030 incidence targets are based on incidences of domestically acquired infections only. Using the Bayesian, negative binomial model, of the four pathogens with a Healthy People 2030 target (*Campylobacter, Salmonella,* STEC, and *Listeria*), no pathogen met the threshold for a decrease in domestically acquired infections, and one met the threshold for evidence of an increase (*Campylobacter*). IRRs for domestically acquired infections were as follows: *Campylobacter* (IRR = 1.07, 95% CrI = 1.01–1.14), *Salmonella* (0.95, 0.88–1.02), STEC (1.14, 1.00–1.30), *Shigella* (0.90, 0.69–1.13), *Yersinia* (2.44, 2.06–2.91), *Vibrio* (1.54, 1.34–1.77), *Listeria* (1.06, 0.92–1.22), and *Cyclospora* (5.30, 2.41–15.18).

^§§§^ Among 2,882 STEC infections, specimens for 2,401 (83%) were cultured; 1,298 (54%) of those cultured yielded an isolate. Of these isolates, 1,293 (>99%) were successfully classified as STEC O157 or STEC non-O157 and 1,187 (91%) had the specific O antigen determined. Therefore, among all STEC infections, 1,293 of 2,882 (45%) infections were classified as STEC O157 or STEC non-O157, and O antigen was determined for 1,187 of 2,882 (41%) infections. Incidences for STEC O157 and overall non-O157 STEC include only a proportion of the overall STEC incidence, because 1,589 STEC infections (55%) were not able to be classified as STEC O157 or STEC non-O157 during 2022, compared with 43% during 2016–2018. Thus, IRRs for STEC O157 and STEC non-O157 partially reflect the increasing proportion of STEC infections with unknown serogroup relative to 2016–2018.

^¶¶¶^ Among STEC isolates classified as O157 or non-O157 (N = 1,293).

**** Incidence rate not calculated.

^††††^ Pathogen for which there is no Healthy People 2030 target.

^§§§§^ For ease of comparison with the Healthy People 2030 incidence target, the reported incidence of domestically acquired *Listeria* infections during 2022 is shown to the second decimal place.

Among 7,032 *Salmonella* infections with positive culture results during 2022, 6,345 isolates (90%) were fully serotyped. The five most common serotypes were Enteritidis (2.7 cases per 100,000 population), Typhimurium (1.6), Newport (1.4), Javiana (0.9), and I 4,[5],12:i:- (0.6), which have been the five most common serotypes each year since 2010. The incidences of two of these serotypes were lower during 2022 compared with those during 2016–2018: Enteritidis (IRR = 0.88, 95% CrI = 0.79–0.97) and I 4,[5],12:i:- (IRR = 0.69, 95% CrI = 0.56–0.86).

Among 2,882 STEC infections, specimens for 2,401 (83%) were cultured; 1,298 (54%) of those cultured yielded an isolate. The O antigen was determined for 1,187 (91%) of the cultured isolates; among those, serogroup O157 was most common (301; 25%), followed by O103 (164; 14%), O26 (155; 13%), and O111 (149; 13%). During 2021, 72 cases of post-diarrheal HUS among persons aged <18 years were reported (0.7 cases per 100,000) (IRR relative to 2016–2018 = 0.96, 95% CrI = 0.82–1.13), including 41 (57%) among persons <5 years old (1.5 per 100,000) (IRR = 0.95, 95% CrI = 0.79–1.18).

The percentage of bacterial infections diagnosed using CIDT increased from 49% during 2016–2018 to 73% in 2022 (Table 2). The percentage of bacterial infections diagnosed using only CIDT increased from 26% during 2016–2018 to 41% in 2022, and, by pathogen, was highest for *Yersinia* (77%), *Vibrio* (56%), and STEC (55%). The overall proportion of reflex cultures that yielded an isolate was similar during 2016–2018 (65%) and 2022 (62%), but decreased for *Salmonella,* STEC, *Shigella*, *Vibrio,* and most markedly for *Yersinia* (from 48% to 24%).

**TABLE 2:** Percentage of bacterial infections diagnosed by a culture-independent diagnostic test, only by a culture-independent diagnostic test, with a reflex culture, and percentage of reflex cultures that yielded an isolate — Foodborne Diseases Active Surveillance Network, 10 U.S. sites,* 2016–2018 and 2022^†^

Pathogen	Infection diagnosed by CIDT,^§^ %		Infection diagnosed only by CIDT,^¶^ %		Positive CIDT with reflex culture,** %		Reflex culture yielded an isolate,^††^ %	
	2016–2018	2022	2016–2018	2022	2016–2018	2022	2016–2018	2022
*Campylobacter*	53	78	36	53	60	54	55	58
*Salmonella*	30	54	9	15	79	86	88	84
STEC	100	100	43	55	88	83	65	54
*Shigella*	49	81	29	52	69	78	58	46
*Yersinia*	69	91	46	77	69	66	48	24
*Vibrio*	45	71	31	56	83	69	38	30
*Listeria*	4	24	0	1	100	100	88	94
**Overall**	**49**	**73**	**26**	**41**	**71**	**70**	**65**	**62**

**Abbreviations:** CIDT = culture-independent diagnostic test; STEC = Shiga toxin-producing *Escherichia coli*.

* Data were obtained from laboratories in Connecticut, Georgia, Maryland, Minnesota, New Mexico, Oregon, Tennessee, and selected counties in California, Colorado, and New York.

^†^ 2022 data are preliminary.

^§^ Includes specimens that had a culture performed, regardless of the result, and those not cultured. The denominator is total infections.

^¶^ Includes specimens that had a negative culture result and those not cultured. The denominator is total infections.

** Specimens with a positive CIDT result that had a culture performed, regardless of the result. Denominator is infections diagnosed by CIDT.

^††^ Denominator is number of specimens having a reflex culture performed.

## Discussion

Many COVID-19 pandemic-related factors influencing enteric disease transmission, detection, and reporting (*3*,*4*) ended by 2022. The incidence of infections caused by pathogens transmitted commonly through food during 2022 generally returned to levels observed during the pre-pandemic period, 2016–2018. Concerted efforts are needed now to implement strategies to reach national prevention targets and lower the prevalence of enteric infections.

This report highlights lack of progress in reducing enteric infection incidence. The incidence of *Salmonella* infections during 2022 was above the Healthy People 2030 target.^§§§^ Also during 2022, the incidence of the most common domestically acquired infections, those caused by *Campylobacter* (17.4 per 100,000 population), was above the Healthy People 2030 target of 10.9. Poultry meat has been the most commonly identified source of *Campylobacter* infections in many countries for many years (*5*) and is also estimated to be the most common U.S. source of *Salmonella* infections (*6*).

Further efforts to reduce contamination during poultry slaughter and processing are needed to reduce the incidence of *Campylobacter, Salmonella,* and other foodborne pathogens (*7*). In 2021, FSIS published new guidelines for poultry slaughter and processing establishments to control *Campylobacter* in raw poultry.^¶¶¶^ Recommendations aim to reduce the incidence of pathogen colonization in birds (e.g., poultry vaccination and use of prebiotics and probiotics) and minimize contamination of poultry water, feed, and bedding. In 2022, FSIS proposed a new regulatory framework to control *Salmonella* in poultry products,**** guided by recommendations from the National Advisory Committee on Microbiological Criteria for Foods. In 2023, FSIS released a proposed notice of determination to declare *Salmonella* an adulterant in not-ready-to-eat breaded and stuffed chicken products.^††††^ Reducing leafy green contamination by improving agricultural water safety, as promoted by FDA^§§§§^ and the Food Safety Modernization Act,^¶¶¶¶^ could also reduce *Salmonella,* STEC, *Listeria,* and other pathogens that cause foodborne illnesses.

In 2022, 73% of infections detected by FoodNet surveillance had a CIDT result (ranging from 24% to 100% by pathogen). These rapid, highly sensitive assays permit prompt clinical diagnoses from a broad range of potential etiologies, enhancing detection of infections that would have otherwise remained undetected. However, CIDT adoption and the routine usage of culture methods has varied by time, pathogen, and market forces (*8*,*9*). These factors and the different sensitivity and specificity of CIDTs complicate the interpretation of surveillance data. Furthermore, having a lower proportion of cases with an isolate obtained by reflex culture limits public health response by reducing the number of isolates having sequenced genomes, which can hinder identification of outbreaks of genetically related infections and the determination of genes coding for antibiotic resistance.

The results of this analysis are subject to at least three limitations. First, the number of reported infections might be undercounted because some ill persons might not seek care, and recommended testing of ill persons might not always be conducted; conversely, false-positive results might cause some overcounting. Second, persons meeting FoodNet criteria for hospitalization or death are included in this report, although underlying reasons for hospitalization or death might be unknown.***** Finally, deaths associated with enteric infections occurring >1 week after specimen collection among patients not hospitalized, and occurring after discharge among those hospitalized (e.g., in hospice care), might have been omitted.

The incidences of infections caused by certain pathogens reported during 2022 were higher than during the prepandemic period 2016–2018, and substantial progress toward Healthy People 2030 objectives was not evident. Prevention measures targeted at reducing food contamination, including the FSIS-proposed *Salmonella* regulatory framework for reducing illnesses from poultry, are needed to mitigate the prevalence of disease and to meet Healthy People 2030 targets. Better understanding of reasons for decreased incidence of foodborne infections during the COVID-19 pandemic (2020–2021) that were not sustained during 2022 could help guide the creation of additional mitigation strategies.

**Summary**

**What is already known about this topic?**

*Campylobacter* and *Salmonella* are the leading causes of bacterial enteric infections transmitted commonly by food. Reported incidence of enteric infections was lower during the COVID-19 pandemic (2020–2021) compared with previous years.

**What is added by this report?**

During 2022, FoodNet identified higher incidences of Shiga toxin-producing *Escherichia coli*, *Yersinia*, *Vibrio*, and *Cyclospora* infections compared with 2016–2018. *Campylobacter, Salmonella, Shigella*, and *Listeria* incidences did not change.

**What are the implications for public health practice?**

Progress in reducing enteric infection incidence was not observed during 2022, as influences of the COVID-19 pandemic subsided. Collaboration among food growers, processors, retail stores, restaurants, and regulators is needed to reduce pathogen contamination during poultry slaughter and to prevent contamination of leafy greens.

## Footnotes

* The FoodNet catchment includes Connecticut, Georgia, Maryland, Minnesota, New Mexico, Oregon, Tennessee, and selected counties in California, Colorado, and New York.

† Reflex culture refers to the process of attempting to grow the identified pathogen in a laboratory culture medium after a CIDT-positive result. Reflex culture practices vary by state and pathogen, and depend on resources, state isolate submission requirements, and specimen viability.

§
http://medrxiv.org/lookup/doi/10.1101/2022.09.14.22279742

¶ Incidence for each year is calculated by dividing the number of infections during that year by the previous year’s U.S. Census Bureau population estimate for the surveillance area. The average during 2016–2018 was calculated by averaging the three incidences for the years 2016, 2017, and 2018.

** Domestically acquired infections are defined as those for which the patient had no history of international travel or unknown travel history. A history of international travel refers to reported international travel during the 30 days before illness began for *Listeria* and *Salmonella* serotypes Typhi and Paratyphi, 14 days before illness began for *Cyclospora*, and 7 days before illness began for other pathogens. Travel information was missing for 24% of infections.

†† Responses that were unknown were included in proportion denominators.

§§ Refers to infections for which the specimen had a culture performed, regardless of the result, and infections for which the specimen was not cultured.

¶¶ To augment HUS case findings by pediatric nephrologists and infection control practitioners, FoodNet staff members annually review hospital discharge data for pediatric HUS cases to validate surveillance reports and identify additional cases by using *International Classification of Diseases, Tenth Revision* (ICD-10) and ICD-11 codes specifying HUS, acute renal failure with hemolytic anemia and thrombocytopenia, or thrombotic thrombocytopenic purpura with diarrhea caused by an unknown pathogen or *E. coli*.

*** 45 C.F.R. part 46.102(l)(2), 21 C.F.R. part 56; 42 U.S.C. Sect. 241(d); 5 U.S.C. Sect. 552a; 44 U.S.C. Sect. 3501 et seq.

††† The most common serotypes associated with *Salmonella* deaths both during 2022 and during 2016–2018 were Enteritidis and Typhimurium. The median age for patients with *Salmonella* deaths during 2022 was 67 years (IQR = 56–76 years) and during 2016–2018 was 68 years (IQR = 53–78 years). During 2022, 10% of *Salmonella* deaths were associated with outbreaks compared with 5% during 2016–2018. In addition, during 2022, 3% of the *Salmonella* deaths with reported travel history were associated with international travel compared with 2% during 2016–2018. The numbers are small, limiting ability to detect differences between reporting periods.

§§§ 11.5 domestically acquired infections per 100,000 population.

¶¶¶
https://www.fsis.usda.gov/sites/default/files/media_file/2021-07/FSIS-GD-2021-0006.pdf

****
https://www.fsis.usda.gov/sites/default/files/media_file/documents/FINAL-Salmonella-Framework-10112022-508-edited.pdf

††††
https://www.fsis.usda.gov/policy/federal-register-rulemaking/federal-register-rules/salmonella-not-ready-eat-breaded-stuffed

§§§§
https://www.fda.gov/food/foodborne-pathogens/leafy-greens-stec-action-plan

¶¶¶¶
https://www.fda.gov/food/guidance-regulation-food-and-dietary-supplements/food-safety-modernization-act-fsma

*****
https://www.cdc.gov/foodnet/surveillance.html
